# Supplementary material for: The effects of genital myiasis on the diversity of the vaginal microbiota in female Bactrian camels
Source: BMC Vet Res. 2022 Mar 5;18:87. doi: 10.1186/s12917-022-03189-5 (PMC8897907; doi:10.1186/s12917-022-03189-5)
Supplement: Supplementary file 5 — Additional file 5. [file 12917_2022_3189_MOESM5_ESM.zip › MPL201709200_16s_yy/Treat1/B13_bdiv/boxplot/boxplot.pdf]

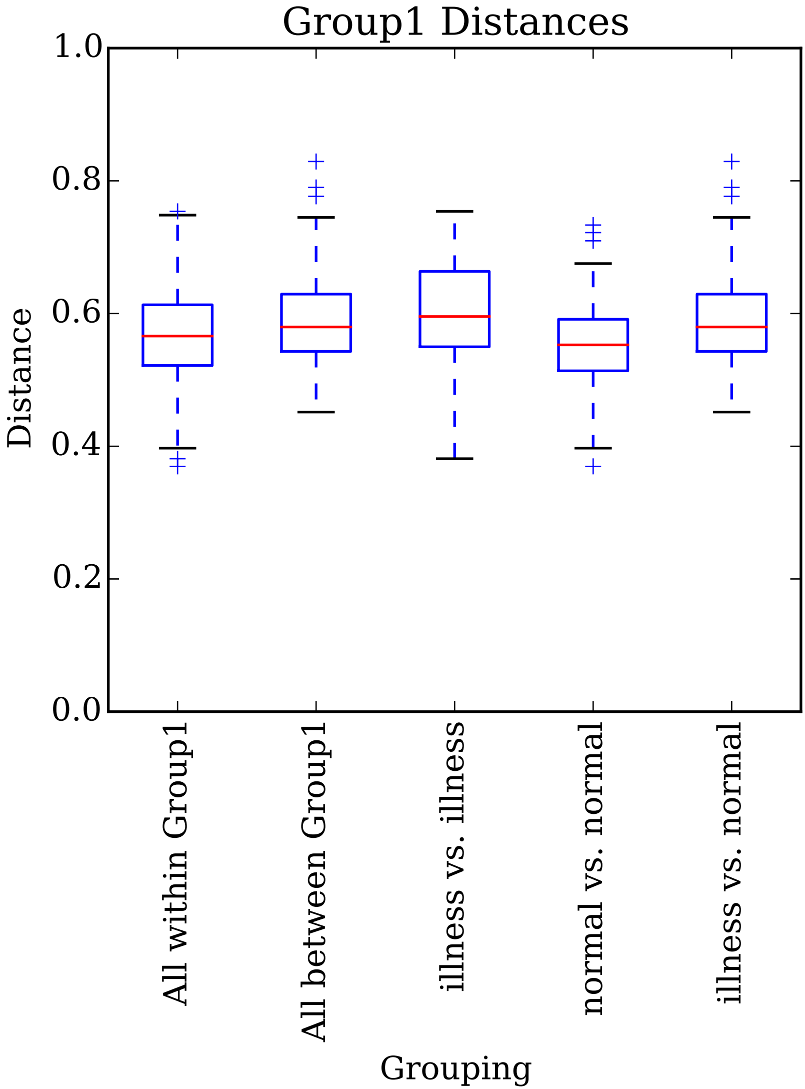
Group1 Distances
1.0
0.8
0.6
0.4
0.2
0.0
Distance
All within Group1
All between Group1
illness vs. illness
normal vs. normal
illness vs. normal
Grouping
